# Supplementary material for: Seminoma and Embryonal Carcinoma Footprints Identified by Analysis of Integrated Genome-Wide Epigenetic and Expression Profiles of Germ Cell Cancer Cell Lines
Source: PLoS One. 2014 Jun 2;9(6):e98330. doi: 10.1371/journal.pone.0098330 (PMC4041891; doi:10.1371/journal.pone.0098330)
Supplement: File S1 — ZIP file containing DMRforPairs output for significant regions. Please start from the html files. (ZIP) [file pone.0098330.s008.zip › figures/41296.pdf]

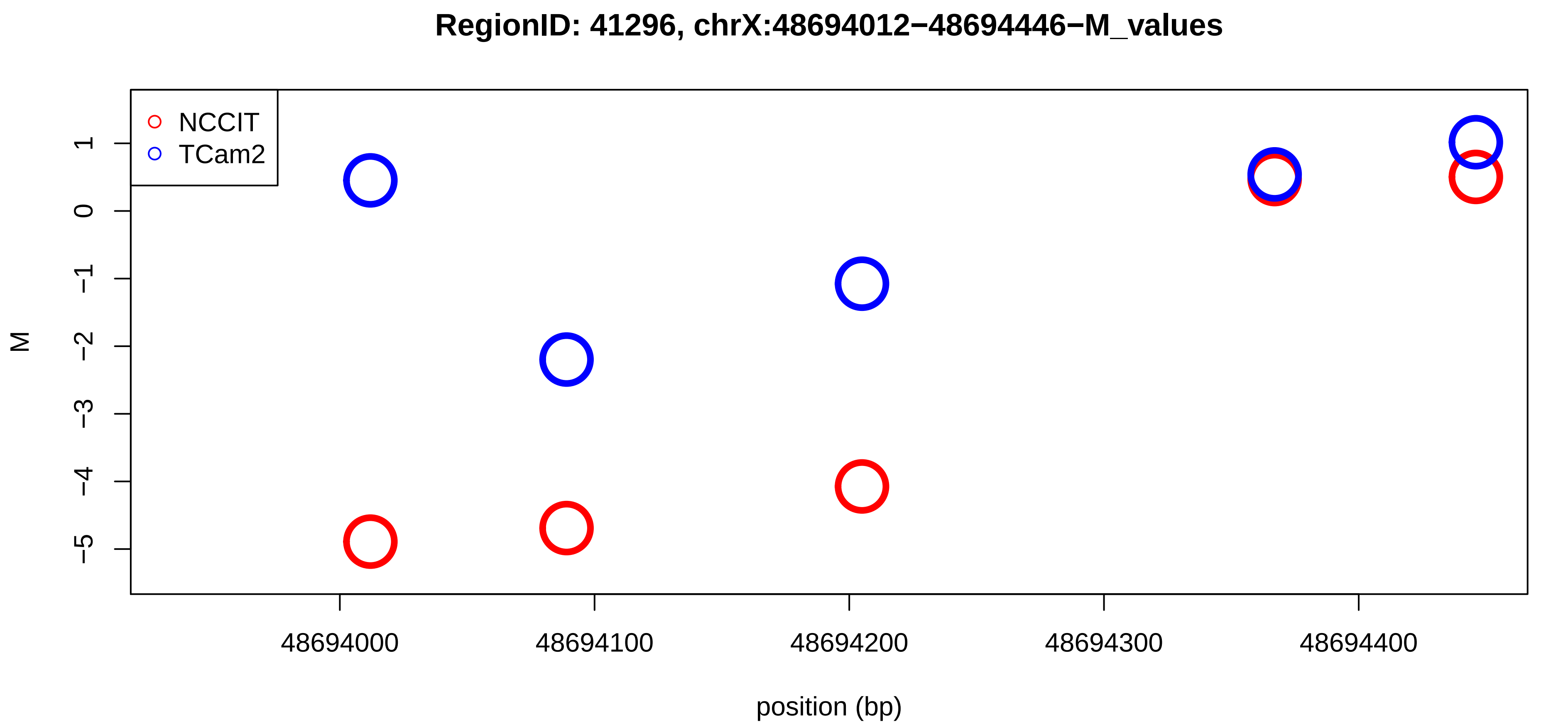
RegionID: 41296, chrX:48694012−48694446−M_values
NCCIT
TCam2
M
position (bp)
48694000
48694100
48694200
48694300
48694400

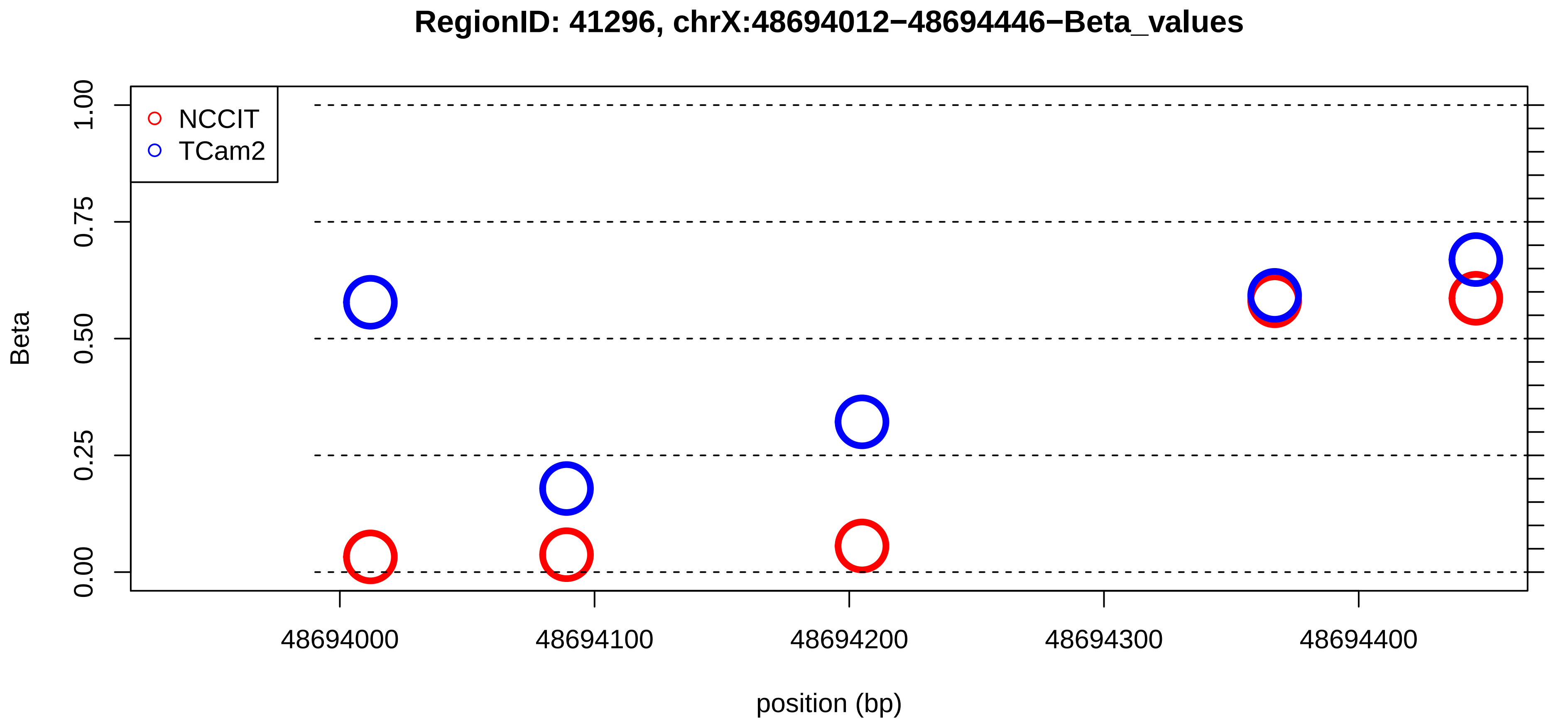
RegionID: 41296, chrX:48694012−48694446−Beta_values
NCCIT
TCam2
Beta
position (bp)
48694000
48694100
48694200
48694300
48694400
